# Insight into Potential Anticancer Activity of Algal Flavonoids: Current Status and Challenges

**DOI:** 10.3390/molecules26226844

**Published:** 2021-11-13

**Authors:** Umme Tamanna Ferdous, Zetty Norhana Balia Yusof

**Affiliations:** 1 Aquatic Animal Health and Therapeutics Laboratory (AquaHealth), Institute of Bioscience, Universiti Putra Malaysia (UPM), Serdang 43400, Selangor, Malaysia; infotama07@gmail.com; 2 Department of Biochemistry, Faculty of Biotechnology and Biomolecular Sciences, Universiti Putra Malaysia (UPM), Serdang 43400, Selangor, Malaysia; 3 Bioprocessing and Biomanufacturing Research Complex (BBRC), Universiti Putra Malaysia (UPM), Serdang 43400, Selangor, Malaysia

**Keywords:** anticancer, antioxidant, flavonoid, microalgae, seaweed

## Abstract

Flavonoids are some of the most precious phytochemicals, believed to be found largely in terrestrial plants. With the advancement of phytochemical research and marine bioprospecting, flavonoids have also been reported by the research of microalgae and macroalgae. High growth rate with minimal nutritional and growth requirement, saving arable land and rich metabolic profile make microalgae an excellent repertoire of novel anticancer compounds, such as flavonoids. In addition, marine algae, especially seaweeds contain different types of flavonoids which are assumed to have unique chemical structures and bioactivities than their terrestrial counterparts. Flavonoids are not only good antioxidants but also have the abilities to kill cancer cells by inducing apoptosis and autophagy. However, the study of the anticancer properties of flavonoids is largely limited to terrestrial plants. This review offers an insight into the distribution of different classes of flavonoids in eukaryotic microalgae, cyanobacteria and seaweeds with their possible anticancer activities. In addition, extraction and purification methods of these flavonoids have been highlighted. Finally, prospects and challenges to use algal flavonoids as anticancer agents have been discussed.

## 1. Introduction

Cancer is ranked as the second-highest cause of death and accounts for about 10 million death in 2020 [1]. Based on the severe negative effects, chemo-resistance, high expenditure and scarcity of novel anticancer compounds, anticancer drug discovery is now more inclined to the investigation of natural sources. About eighty percent of all approved chemotherapeutics and fifty percent of all drugs are derived from natural origin. Natural drugs are accepted as a safer option than synthetic drugs because of their presence in the human diet and broad availability. Furthermore, natural drugs have reduced side effects and the potential to target various tumorigenesis-related signaling pathways. Considering all of these advantages, natural products research is burgeoning to search for novel anticancer compounds not only from terrestrial plants and microorganisms but also from marine organisms [2]. Marine organisms are offering a huge array of pharmaceutically important natural products that can be used to treat different kinds of human diseases, especially cancer. The chemical structures of marine-derived bioactive compounds are unique and highly diversified. Marine organisms show novel molecular scaffolds than terrestrial products [3]. Currently, a total of 14 marine-derived drugs are available on the market and 9 of them are used for cancer therapy. Another 19 compounds from the marine organisms are in different phases of cancer clinical trials. The anticancer compounds derived from marine compounds mainly come from mollusk/cyanobacterium, sponge, tunicate, bacterium, fungus and sea animals. Mollusk/cyanobacteria account for 64% of anticancer drugs [4]. Four anticancer drugs are already on the market from marine cyanobacteria species. Several other anticancer drug candidates from cyanobacteria species are in phase I–III clinical trials and also in preclinical trials. Eukaryotic microalgae are also under-exploration for discovering anticancer drug leads or fractions, as many of these eukaryotic microalgae are traditionally used as food and feed [5]. Marine microalgae are rich sources of pharmaceutically important bioactive compounds, like carotenoids, phenolics, flavonoids, fatty acids, alkaloids and other compounds. The advantages of using microalgae in drug discovery include their short generation time, metabolic plasticity, no competition for the arable land, their growing capacity irrespective of any season and less amount of special nutritional requirement to grow [6]. In terms of bioactivity and amount of phytochemicals, microalgae are richer than terrestrial plants [7]. For example, microalgae can produce more carotenoids, such as lutein, astaxanthin, than higher plants. Moreover, these carotenoids can be harvested in a less labor-intensive way and with low cost, in comparison to higher plants [8]. In addition, marine microalgae are assumed to produce unique metabolites with extensive chemical diversity, as they are facing extreme environments more often, like high salinity or temperature [3]. Microalgae and macroalgae are also rich sources of flavonoids [9].

**Figure 1:**
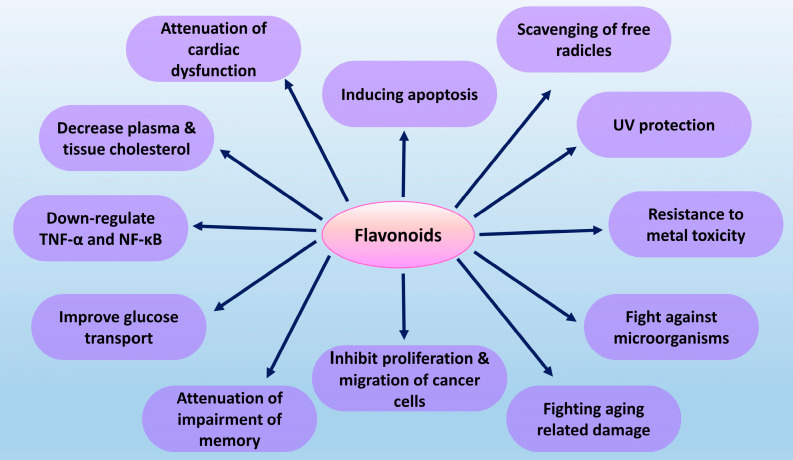
Biological function of flavonoids [11,12].

Flavonoids are the most common plant polyphenols that are incorporated into our diet abundantly. Their complex molecular structures are related to biological functions in the human body. Plant flavonoids are extensively studied for their anticancer activities, along with other biological functions (Figure 1). Flavonoids derived from higher plants are successful in killing cancer cells and some of them are in different phases of clinical trials. A total of twenty-two Phase II clinical trials and one Phase III clinical trial investigated flavonoids alone or combined with other therapeutics either on hematopoietic/lymphoid or solid tumors. The most commonly tested flavonoid was Flavopiridol [10]. Though plant-derived flavonoids are under developing for new cancer drug, algal flavonoids are still limited to detection. No remarkable data is evident for their anticancer activity. However, their high flavonoid content and the prospect of having a unique flavonoid class make them promising sources of these compounds as anticancer agents (Table 1). This review, therefore, highlights the presence and quantity of flavonoids in different algal sources and possible anticancer activities.

## 2. Flavonoids

**Figure 2:**
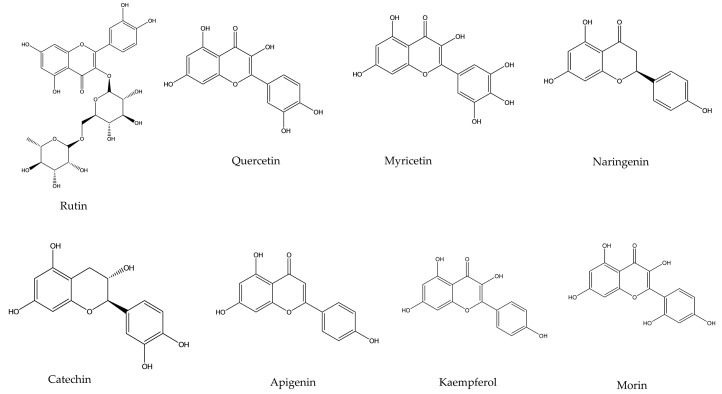
Chemical structure of commonly found flavonoids.

Flavonoids are a large group of polyphenolic metabolites which is widely dispersed throughout the plant species. Among many of the plant pigments, flavonoids are classed as edible. They are derived from derivatives of 2-phenyl-benzo-γ-pyrone [41]. Flavonoids have a common flavan structure that is a 15-carbon phenylpropanoid chain (C6-C3-C6), which is assembled into two aromatic rings and linked with another O_2_ containing pyrene ring. This basic structure is changed through oxidation and also modified in substituents to the pyrene ring that leads to the formation of other flavonoid classes [42]. Two aromatic rings are condensed to form a chalcone. Cyclization of chalcone forms flavanone which is the primary compound for the synthesis of other classes of flavonoids. Flavonoids are categorized into six different types. Flavanols (catechin, epicatechin, epigallocatechin), Flavanones (naringenin and hesperetin), Flavonols (quercetin, kaempferol or myricetin), Isoflavones (genistein and daidzein), Flavones (apigenin and luteolin) and Anthocyanidins are the groups (Figure 2) found in plant and algae [41].

## 3. Algal Flavonoids

Flavonoids are widespread in algae and the concentration of flavonoids can be further increased through different manipulation or enhancement techniques. For example, increased salt concentration has a significant effect on the accumulation of phenolics and flavonoids. The growth of cyanobacterial species *Plectonema boryanum*, *Anabaena doliolum* and *Oscillatoria acuta* were decreased when treated with high NaCl concentration but the accumulation of rutin was increased [13]. Metal stress can also help in the accumulation of flavonoids in microalgae. *Dunaliella tertiolect* has been reported to have increased catechin and epicatechin with an augmented amount of copper and these flavonoids are thought to be acted as a protector of microalgae cells from metal toxicity. The extracts containing these flavonoids also showed antioxidant activity [19]. With the increased concentration of nitrate and growth factor L-phenylalanine, *Spirulina maxima* showed accumulation of a higher amount of flavonoids, such as quercetin and kaempferol. The presence of these flavonoids along with other phenolics gave antioxidative protection against lipid peroxidation of hepatic microsomes in rats which were induced by carbon tetrachloride, an oxidizing agent. The results were comparable to the commercial antioxidant BHT and BHA [43]. Yadavalli et al. also reported the accumulation of flavonoids, like quercetin and catechin, in *Chlorella vulgaris* under nitrate-stressed-condition with supplementation with L-phenylalanine. Depletion of nitrate in the media upregulates the synthesis of flavonoids and the rate has been accelerated after the addition of L-phenylalanine due to its direct link in the flavonoid synthesis pathway [44]. In *Leptolyngbya* sp., catechin, apigenin, naringenin, luteolin and luteolin-7-glucoside were found to be the most common flavonoids. Among them, naringenin was the highest, 4.1 ± 0.01 mg/g [27].

Singh et al. investigated twenty terrestrial cyanobacteria species and detected three main flavonoids namely, quercetin, kaempferol and rutin. Rutin was found at highest level in *Microcheate tenera* (29.4 ± 0.7 µg/g) and the lowest amount in *Chroococcus* sp. (1.4 ± 0.3 µg/g) whereas quercetin was found mostly in *Nostoc ellipsosporum* (23.8 ± 1.03 µg/g) but least amount was detected in *Westiellopsis prolific* (3.7 ± 0.5 µg/g). Kaempferol was found in the highest amount in *M. tenera* and *W. prolific,* whereas *Phormidium tenue* was the cyanobacteria that produced the lowest amount of kaempferol [14].

Goiris et al. investigated the major flavonoid contents in different microalgae species. The frequently found flavonoids in the studied microalgae were naringenin, apigenin, luteolin, genistein, dihydroquercetin, daidzein, quercetin, kaempferol, catechin, epicatechin and proanthocyanidins. Among them, apigenin was detected in all the microalgae species, namely, *Phaeodactylum tricornutum*, *Diacronema lutheri*, *Porphyridium purpureum*, *Haematococcus pluvialis*, *C. vulgaris*, *T. suecica* and *Arthrospira platensis*. *H. pluvialis* contained most of the flavonoids tested, quercetin, dihydroquercetin, kaempferol, dihydrokaempferol, naringenin, apigenin, luteolin, genistein and daidzein [9].

Seaweeds, especially red and brown algae, are often considered a superfood. Different seaweed species are consumed as food and supplements worldwide. Likewise, eukaryotic microalgae *Chlorella* spp. and cyanobacteria *Spirulina* sp. are also now quite popular in the market of food supplements. These edible macro- and microalgae are rich in polyphenols, where one-third is phenolic acids and the rest two-thirds are flavonoids. A study showed that flavonoid, particularly epicatechin, was the most frequently found phenolic compound in commercial algal food products, which was detected in each red and brown seaweeds, as well as in *Chlorella pyrenoidosa* and *Spirulina platensis*. Other than this flavonoid, catechin gallate, epicatechin gallate, epigallocatechin, epigallocatechin gallate and pyrocatechol was measured in those algae. However, the most interesting thing is that the antioxidant capacity of these water-soluble compounds (ACW) of brown algae, *Eisenia bicyclis*, was found more than bilberry, strawberry and kiwi [32]. A very comprehensive study was done by Yoshie et al., where twenty-seven Japanese seaweeds from red, green, brown types were evaluated for their flavonoids contents. They found mainly catechin, epicatechin, epigallocatechin and epigallocatechin gallate in different seaweed species. From their study, catechin was found in most of the red and brown species but detected in the highest in *Acetabularia ryukyuensis*, which is a green alga [45]. In another study, they found rutin, quercetin, myricetin and two other unique flavonoids, hesperidin and morin. Morin was found in each species. Similarly, hesperidin was also detected in most of the seaweeds, even more than rutin, quercetin and myricetin. The highest amount of hesperidin was detected in *A. ryukyuensis*, *Gracilaria texorii* and *Gracilaria asiatica* [25].

### 3.1. Flavonol

#### 3.1.1. Rutin

Flavonoid glycoside, rutin (3′,4′,5,7-tetrahydroxy-flavone-3-rutinoside)) is a dietary flavonoid and frequently found in more than seventy plant species. It is also known as vitamin P. Rutin was found in the aerial part of *Ruta graveolens* and hence, the name was derived from this plant [24]. Rutin is widely found in cyanobacterial species. *Microcheate tenera* (29.4 ± 0.7 µg/g fresh weight), *Mastigocladus laminosus* (13.4 ± 0.4 µg/g fresh wt), *Anabaena doliolum* (12.8 ± 0.41 µg/g fresh wt), *Calothrix geitonos* (12.0 ± 0.4 µg/g fresh wt) were reported to contain rutin. This cyanobacterial rutin exhibited higher radical scavenging activity compared to BHT and α-Tocoferol [14]. *Plectonema boryanum*, *Hapalosiphon intricatus* and *Oscillatoria acuta* also contain rutin but in lesser amount [13]. *Dunaliella tertiolecta* also produces rutin but in high ferric concentration, 900 nmol/L and low copper concentration, 315 nmol/L [19].

Rutin kills cancer cells through an increased level of ROS that causes oxidative stress in the cells. It can inhibit PI3K/Akt and Ras/Raf/MAPK signaling pathways that ultimately cause cell cycle arrest and, finally, apoptosis. Upregulation of Bax and downregulation of MMP-2 and Bcl-2 by rutin can also lead to apoptosis through caspase-3 activation. In the animal model, rutin can effectively reduce doxorubicin-induced neurotoxicity and nephrotoxicity [46].

#### 3.1.2. Quercetin

Quercetin, a pentahydroxyflavone, is widely found in vegetables and fruits, like apples, grapes, berries, citrus fruits, onions, broccoli and green tea. Quercetin can be found in algae, *Nostoc ellipsosporum* (23.8 ± 1.03 µg/g fresh weight), *Limnothrix obliqueacuminata* (12.4 ± 0.43 µg/g fresh wt), *Microcheate tenera* (18.4 ± 0.85 µg/g fresh wt). In addition, they can be found in *Hapalosiphon fontinalis*, *Scytonema simplex*, *Calothrix brevissima*, *Limnothrix* sp. and *Phormidium tenue* in almost similar amounts (around 11 µg/g fresh wt). Moreover, quercetin showed more potent anti-oxidative activity (IC_50_ = 4.71 ± 0.49 µg/mL) than α-tocoferol and commercial antioxidant BHT, which was revealed by DPPH radical scavenging assays [14]. It has been reported that quercetin has higher antiradical activity when compared to other phenolics, like rutin and catechol [47].

Quercetin also have strong anticancer activity. Quercetin has been reported to arrest the cell cycle by releasing p53 that augments p21, GADD45 and Bax expression, as well as impedes CDK2, cyclin A and B activity. In addition, it hindered the migration of cancer cells through upregulation of the expression of E-cadherin and downregulation of *N*-cadherin, Vimentin, MMP-2, -7 and Snail-dependent Akt activation pathway. On top of that quercetin can induce autophagic cell death by modulating LC3Ι. Quercetin induced apoptotic and autophagic cell death in the mouse model as well [48]. Furthermore, quercetin potentiated the efficacy of sorafenib in lower doses in thyroid cancer therapy by increasing the expression of E-cadherin and decreasing the expression of *N*-cadherin [49].

#### 3.1.3. Kaempferol

Kaempferol (3,5,7-trihydroxy-2-(4-hydroxyphenyl)-4H-1-benzopyran-4-one) is classed among flavonol and is mostly found in cabbage, broccoli, beans, tomatoes and berries [21]. Kaempferol has been reported to be found in *Nostoc* and *Anabaena*, which is 1.08 and 1.23 mg/g of dry wt of the extract with good antioxidant property [15]. Marine microalgae, *Nannochloris* sp. produced kaempferol in higher amounts, 12.10 ± 1.32 µg/g [22]. Kaempferol is found mostly in *M. tenera* (7.8 ± 0.7 µg/g fresh wt), *Westiellopsis prolific* (7.8 ± 0.46 µg/g fresh wt), *H. fontinalis* (4.8 ± 0.6 µg/g fresh wt), *Cylindrospermum* sp. (3.9 ± 0.36 µg/g fresh wt). This flavonoid showed stronger antioxidant activity with less IC_50_ than α-Tocoferol and BHT [14].

Kaempferol can induce apoptosis in ovarian cancer cells through increased expression of apoptotic proteins, caspase-3, -8, -9, Bax, or by stimulating death receptors/FADD/Caspase-8 pathway. In these cancer cells, it stopped the progression of the cell cycle in G2/M phase by inducing the Chk2/Cdc25C/Cdc2 and Chk2/p21/Cdc2 pathways [50,51]. In pancreatic cancer cells, it induced apoptosis by activating tissue transglutaminase (TGM2) mediated Akt/mTOR signaling pathway that increased the ROS production in the cells [52].

#### 3.1.4. Morin

Morin (3,5,7,2’,4’-pentahydroxyflavone) is a unique flavonoid in all types of algae species and the highest amount was reported in green seaweed, *Caulerpa serrulata* with an amount of 3730 ± 23 µg/g fresh weight. Morin was found in other species of seaweeds like, *Monostroma nitidum*, *Caulerpa racemose*, *Caulerpa serrulata*, *Undaria pinnatifida*, *Eisenia bicyclis*, *Ishige okamurae*, *Laminaria religiosa*, *Porphyra yezoensis* and *Chondrus verruscosus* [25].

Morin exerted anticancer activity against chronic myeloid leukemia via downregulation of the PI3K/AKT signaling pathway and also miR-188-5p which led to apoptosis [53]. In triple-negative breast cancer cells (MDA-MB-231), morin exerted anti-proliferative activity by arresting cells at S and G2/M phase, via upregulation of the ERK/p21 signaling pathway and downregulation of the FOXM1 signaling, which reduced cyclin A2 and cyclin B1 [54]. Another anticancer activity of morin is the downregulation of Glut 1 expression that restricts the entry of glucose into the cells. As glucose is the main nutrient, blocking its uptake leads to mitochondria-mediated apoptosis [55].

### 3.2. Flavanol

#### 3.2.1. Catechin

Catechin, a flavan-3-ol, is mainly found in *Camellia sinensis* and *C. assumica*. Catechin is divided into eight different classes [28]. Catechin has been detected in *Euglena cantabrica* (71.4 µg/g of dry weight) and these flavonoid-containing extracts have radical scavenging activity [29]. It was also found in *Dunaliella tertiolecta*, even when exposed to low copper concentration, up to 315 nmol/L [19]. Seaweed, *Porphyra tenera* produced a high amount of catechin than the microalgae, which was measured as 128.8 ± 2.9 µg/g [32].

Catechin can kill human glioma (U87MG) cells in a concentration-dependent manner while showing lower cytotoxicity in normal astrocytes. It can inhibit cell proliferation by blocking cells in G2/M phase of the cell cycle and also by inhibiting MAPK/ERK signaling pathway. Moreover, catechin can cause autophagy-induced glioma cell death by forming autophagosomes and autophagic vacuoles and triggering increased expression of LC3II and decreased expression of p62 [56]. Nanohybrid formulation with catechin (50 µg/mL) inhibited human melanoma (WM266-4) cells proliferation and new blood vessel formation in zebrafish xenotransplants [57].

#### 3.2.2. Epicatechin

Epicatechin, another flavanol, is subclassed into catechin and was isolated from the pith of the palm *Metroxylon sagu.* Epicatechin is abundant in green tea and black tea, berries, red wine and cacao. Epicatechin is also found in algae, such as *Euglena cantabrica* but in small amounts (7.1 µg/g of dry weight) [19]. In addition, epicatechin was found in edible macroalgae, *Porphyra tenera* but the highest amount was found in *Spirulina platensis,* at concentration of 27.5 ± 1.3 µg/g [32]. Along with other bioactivities, epicatechin exerts anticancer activity. For example, epicatechin can kill breast cancer (MDA-MB-231 and MCF-7 cells,) cells in a concentration-dependent manner with an IC_50_ of 350 µM. It can induce apoptosis through DNA fragmentation, augmentation of the expression of pro-apoptotic proteins (Bad and Bax) and an increased level of ROS [58]. Epicatechin can act as a restorative agent which is assumed to mitigate negative side effects of chemotherapy drug, bleomycin and helps improving lung damage which in turn enhances the quality of life of the patient. In the animal model, this flavanol reduced the negative effects of bleomycin by alleviating oxidative stress, inflammation and fibrosis [59].

#### 3.2.3. Epigallocatechin-Gallate (EGCG)

Epigallocatechin-gallate (EGCG) is also classed among catechin and is also mostly found in green tea. An edible seaweed *Undaria pinnatifida* (also called Wakame in Japanese) has been reported to produce a high amount of EGCG (7.5 ± 0.1 µg/g) and also *P. tenera* produced this flavonoid but in less amount, 4.0 ± 0.1 µg/g [32]. Among the catechins, EGCG is the most potent anticancer compound. It shows chemo-preventive action against twenty-four different types of cancer [60]. EGCG can inhibit cell proliferation, angiogenesis and metastasis. Moreover, it can kill cancer cells through apoptosis and autophagy [61]. To exert anticancer activity, EGCG modulated different signaling pathways like JAK2/STAT3/AKT, VEGF/VEGFR. TGF/SMAD, Wnt/β-catenin, Notch pathway and TRAIL-mediated pathway [62].

### 3.3. Flavone

#### 3.3.1. Apigenin

Apigenin (4′,5,7-trihydroxyflavone) is a flavone and widespread in different plant species. A high amount of apigenin was found in the biomass methanol extract of cyanobacteria *Leptolyngbya* sp., which was 0.4 ± 0.02 mg/g and this methanol extract showed the highest radical scavenging activity [27].

Apigenin can kill different kinds of cancer cells. In breast cancer cell line MCF-7, apigenin can induce apoptosis through increased ROS production and DNA fragmentation. In addition, it can enhance the expression of p53, Bax/Bcl-2 ratio, caspase proteins and thus, influencing the cleavage of PARP. Apigenin can arrest cells at G2/M phase as well [63]. In cisplatin-resistant colon cancer cells HT-29, apigenin can augment the expression of Beclin-1 and LC3-II and downregulate the expression of p62 which leads to autophagic death. It can also induce apoptosis by increasing Bax expression while downregulating Bcl-2 expression in the same cell line [64].

#### 3.3.2. Dimethoxyflavon

Marine microalgae *Phaeodactylum tricornutum*, *Nannochloris* sp. and *Tetraselmis suecica* from Mediterranean Morocco have been reported to produce dimethoxyflavon. Among them, *P. tricornutum* has the highest amount of dimethoxyflavon, 28.38 ± 2.90 µg/g [22].

In a study, dimethoxyflavone induced apoptosis in endometrioma cells by inhibiting PI3K/AKT and ERK1/2 signaling pathways, activating ER-stress response proteins and MAPK proteins, JNK and p38, increasing the production of ROS and calcium in high levels which led to disruption of mitochondrial membrane potential while inactivating the PI3K/MAPK pathways [65].

### 3.4. Isoflavone

#### Genistein

Genistein [5,7-dihydroxy-3-(4-hydroxyphenyl) chromen-4-one] is classed among isoflavones. It was first discovered in *Genista tinctoria* and thus, named after this plant. It is found in leguminous plants, mainly in soybean. However, genistein is not quite common in algae species. Goiri et al. reported the highest level of genistein in *Phaeodactylum tricornutum*, 1.42 ± 0.14 ng/g [9].

Cancer cells are affected by genistein via apoptosis induction, cell cycle arrest, inhibition of metastasis and angiogenesis. To induce apoptotic death, genistein hindered NF-κB pathways and upregulates pro-apoptotic proteins like, Bax, Bad and Bak, as well as cyt c release which in turn caused caspase-dependent apoptosis. Genistein also induced calpain which is a Ca^2+^ dependent protein that is responsible for cleaving Bax and Bid, rendering apoptotic cell death. Genistein can impede the cell cycle by modulating Ras/MAPK/activator protein-1 and downregulating the expression of Cdk1, cyclin B1 and Cdc25C. Moreover, genistein inhibited angiogenesis through reduced expression of VEGF, MMP-2/9 and JNK, p38, PTK/MAPK pathways [37].

### 3.5. Flavanone

#### Hesperidin

Hesperidin (3′,5,7-trihydroxy-4′-methoxyflavanone-7-rhamnoglucoside) is a bioflavonoid that is mainly found in citrus fruits, especially orange and lemon [39]. Hesperidin is mostly found in red algae. In a study of Japanese seaweed flavonoid content, *Gracilaria texorii* was found to have the highest amount of hesperidin, 119,000 ± 1800 µg/g fresh weight [25].

Hesperidin exerts its anticancer activity against different cancer cells. In MDA-MB231 breast cancer cells, it can inhibit metastasis of these cells by downregulation of the expression of programmed death-ligand 1 (PD-L1) with reduced expression of Akt and NF-κB signaling and also decrease in the expression of MMP-9 and MMP-2 [66]. A study showed that co-administration with imatinib mesylate, hesperidin potentiated the drug action on the imatinib-resistant breast cancer cells and also with less negative effects. It downregulated the expression of the multidrug-resistant (MDR-1) gene, thus, overcome the drug resistance. Hesperidin upregulated Bax/Bcl-2 and caspase-3 expression to induce apoptosis. In addition, it protected heart tissue which was revealed through the reduced serum enzymes LDH and SGOT [67].

## 4. Prospects and Limitations

Flavonoids are an excellent reservoir of biological activities that can be exploited to treat different medical conditions, for instance, diabetes, cardiovascular dysfunction, ocular diseases, aging problem and neurological complications. Some clinical trials also proved the efficiency of using flavonoids in different kinds of health-related problems. However, utilizing flavonoids in the drug discovery process have some major concerns. Flavonoids present in a very small amount in plants or algae, from µg to mg per kg of biomass. Moreover, this small amount is mostly found in complex with other bioactive compounds, which renders it difficult to determine the actual source of the pharmacological effect. Purification and identification of these flavonoids are a multistage, expensive and time-consuming process. In addition, their high lability and being prone to chemical alteration can lead to degradation during the purification process. Poor bioavailability is another problem for flavonoids as a drug. However, with novel optimization techniques, multiplex purification systems and metabolic engineering, flavonoids can be extracted in a high amount from natural sources. However, another most important problem for plant flavonoids is that a regular and high amount of extraction from plant species may cause the elimination of useful plant species or may risk food security [68]. Algae, as a potential source of flavonoids, offer a suitable solution for this problem.

Microalgae give several benefits over higher plants in the production of high-value products. The fast and foremost benefit of microalgae is their high photosynthetic efficiency than terrestrial plants, which is linked to their fast growth rate and high yield per unit dry biomass than plants. Moreover, microalgae can be grown in ranges of water media; saltwater, freshwater and even wastewater. Industrial effluent or aquaculture wastewater can be an alternative source of media for the economic production of microalgae [69]. Moreover, microalgae are now grown in large photobioreactors which limits vast land-use and contamination risk. Microalgae and seaweed farming don’t have any impact on food security and the environment, as no chemical fertilizer is used in the cultivation process. Specialized photobioreactors provide optimum and evenly distributed illumination to the microalgae culture which is important for a high production rate. Though this sophisticated bioreactor is a bit expensive. Efficient and low-cost harvesting is another challenge to get high-value compounds from microalgae. In recent years, membrane bioreactors are in use for harvesting microalgae which is a cheaper but efficient harvesting alternative. The system called magnetically induced membrane vibration makes this harvesting process more easier and cost-effective [70]. Therefore, to get benefit from these excellent species, a suitable and effective separation technique along with extensive anticancer study is warranted for algal flavonoids.

## 5. Conclusions

Microalgae and seaweeds are both suitable sources of different classes of flavonoids. However, the anticancer study from such valuable sources is overlooked. Though studies predicted that the total flavonoid contents found in the microalgae species is responsible for the antitumor activity in various cell lines, further purification and identification have not been done yet. More emphasis should be put on the isolation of specific flavonoids from algal sources and their bioactive properties. Moreover, seaweed and marine microalgae may possess unique flavonoid types, which can further be revealed through marine bioprospecting. Thus, algae can become an alternative source of flavonoids with anticancer activity.

## Figures and Tables

**Table 1:** Sources of algal flavonoids and their possible anticancer activity.

Subclass	Flavonoids	Algae Source	Amount	Possible Bioactivity	Reference
Flavonol	Quercetin	*Nostoc ellipsosporum*	23.8 ± 1.03 µg/g fresh wt	Anticancer, Antioxidant, Antimicrobial, Antidiabetic, anti-inflammatory, Neuroprotective, Hepatoprotective	[13,14,15,16,17,18]
		*Microcheate tenera*	18.4 ± 0.85 µg/g fresh wt		
		*Limnothrix* *obliqueacuminata*	12.4 ± 0.43 µg/g fresh wt		
		*Hapalosiphon fontinalis*	11.7 ± 0.66 µg/g fresh wt		
	Myricetin	*Dunaliella tertiolecta*	6.5 ± 0.6 attomol/cell	Anticancer, antidiabetic, anti-infectious, antioxidant, anti-inflammatory, anti-obesity, neuroprotective	[19,20]
		*Tubinaria ornata*	346 ± 3.4 µg/g		
	Kaempferol	*Nannochloris* sp.	12.10 ± 1.32 µg/g	Anticancer, Antioxidant, Anti-inflammatory, Antidiabetic, Neuroprotective	[14,15,21,22,23]
		*Microcheate tenera*	7.8 ± 0.7 µg/g fresh wt		
		*Nostoc ellipsosporum*	4.3 ± 0.6 µg/g fresh wt		
		*Hapalosiphon fontinalis.*	4.8 ± 0.6 µg/g fresh wt		
		*Westiellopsis prolific*	7.8 ± 0.46 µg/g fresh wt		
	Rutin	*Microcheate tenera*	29.4 ± 0.72 µg/g	Anticancer, antioxidant, antimicrobial, antidiabetic, anti Inflammatory, neuroprotective, cardioprotective, hepatoprotective, nephroprotective	[13,14,15,19,24]
		*Dunaliella tertiolecta*	2.8 ± 0.3 attomol/cell		
		*Hapalosiphon intricatus*	9.61 µg/g fresh wt		
		*Calothrix geitonos*	12.0 ± 0.4 µg/g fresh wt		
		*Mastigocladus laminosus*	13.4 ± 0.46 µg/g fresh wt		
		*Lyngbya* sp.	8.8 ± 0.6 µg/g fresh wt		
		*Phormidium*	8.8 ± 0.6 µg/g		
		*Nostoc* sp.	4.52 mg/g		
	Morin	*Caulerpa serrulata*	3730 ± 23 µg/g	Anticancer, antioxidant, anti-microbial, antidiabetic, neuroprotective, anti-arthritis, anti-inflammatory, nephroprotective, cardio protective, hepatoprotective	[25,26]
Flavanol	Catechin	*Porphyra tenera*	128.8 ± 2.9 µg/g	Anticancer, Antioxidant, Antimicrobial, Anti-allergenic, anti-inflammatory, UV protection activity	[19,27,28,29,30,31]
		*Spirulina platensis*	22.7 ± 2.3 µg/g		
		*Nannochloris* sp.	33.47 ± 3.14 µg/g		
		*Dunaliella tertiolecta*	36.1 ± 0.8 attomol/cell		
		*Euglena cantabrica*	71.4 µg/g		
		*Leptolyngbya* sp.	2.6 ± 0.2 mg/g		
		*Anabaena* sp.	35.19 µg/g DW		
	Epicatechin	*Dunaliella tertiolecta*	24.4 ± 0.1 attomol/cell	Anticancer, Antioxidant, Antidiabetic, anti-inflammatory, Cardio-protective, Neuroprotective	[19,29,30]
		*Spirulina platensis*	27.5 ± 1.3 µg/g		
		*Porphyra tenera*	16.4 ± 0.7 µg/g		
		*Hizikia* *fusiformis*	8.2 ± 0.1 µg/g		
	Epigallocatechin-gallate	*Undaria pinnatifida*	7.5 ± 0.1 µg/g	Anticancer, Antioxidant, Antimicrobial, Anti-allergic, Antidiabetic, anti-inflammatory, Cardio-protective, Neuroprotective	[32,33]
		*Porphyra tenera*	4.0 ± 0.1 µg/g		
Flavone	Apigenin	*Leptolyngbya* sp.	0.4 ± 0.02 mg/g	Anticancer, antidiabetic, neuroprotective, anti-arthritis, anti-depressant, anti-inflammatory	[27,34]
	Luteolin-7-glucoside	*Diacronema lutheri*	0.8 ± 0.06 ng/g	Anticancer, anti-inflammation, anti-allergy and antioxidant	[9,27,35]
		*Leptolyngbya* sp.	0.4 ± 0.01 mg/g		
	Dimethoxyflavon	*Phaeodactylum tricornutum*	28.38 ± 2.90 µg/g	Anticancer, antifungal	[22,36]
		*Tetraselmis suecica*	19.01 ± 1.58 µg/g		
Isoflavone	Genistein	*Phaeodactylum tricornutum*	1.42 ± 0.14 ng/g	Anticancer, Antioxidant, Antimicrobial, Antidiabetic, Cardioprotective	[9,37]
	Daidzein	*Phaeodactylum tricornutum*	5.9 ± 0.6 ng/g	Anticancer, antidiabetic, Anti-Osteoporosis, anti-aging, antioxidant, anti-microbial, anti-inflammatory	[9,38]
Flavanone	Naringenin	*Leptolyngbya* sp.	4.1 ± 0.01 mg/g	Anticancer, antioxidant, antimicrobial, anti-inflammatory, antiadipogenic, anti-diabetic, cardioprotective, eye-protective	[9,12,27]
		*Diacronema lutheri*	0.60 ± 0.06 ng/g		
	Hesperidin	*Gracilaria texorii*	119000 ± 1800 µg/g	Anticancer, anti-allergic, anti-oxidant and anti-inflammatory	[25,39]
Flavanonol	Dihydroquercetin	*Haematococcus pluvialis*	1.6 ± 0.16 ng/g	Anticancer, antioxidant, anti-bacterial	[9,30,40]

## Data Availability

The data presented in this study are available on request from the corresponding author.
